# Treatment of Two-column Acetabular Fractures by Double Extrapelvic Approach: Three Clinical Cases

**DOI:** 10.1055/s-0041-1729934

**Published:** 2021-08-13

**Authors:** João Boavida, Paulo Gil Ribeiro, Paulo Costa, Catarina Quintas, Diogo Lino Moura, António Figueiredo

**Affiliations:** 1 Serviço de Ortopedia, Centro Hospitalar e Universitário de Coimbra EPE, Coimbra, Portugal

**Keywords:** acetabulum, fractures, bone, pelvis

## Abstract

Fractures of two columns of the acetabulum according to the Letournel classification are among the most common in frequency, indication and surgical complexity. These are mainly the result of lateral compression mechanisms and are characterized by originating a disconnected acetabulum from the axial skeleton. Its surgical treatment may include: isolated anterior or posterior approach; combined, at the same surgical time or not; or broad approaches. The authors present another surgical option with association of the Kocher-Langenbeck pathway with the iliac crest approach simultaneously and in the same positioning (lateral decubitus) based on the first three clinical cases performed and their clinical and imaging results. In addition to the presentation of the cases, a description of the three characteristic fragments of this type of acetabular fractures, the approach pathway, and the reduction sequence performed are made. From the results obtained and the associated advantages, the authors believe that the addition of the iliac crest approach to the Kocher-Langenbeck pathway may be a very attractive option to consider in the surgical treatment of properly selected fractures of two columns of the acetabula.

## Introduction

Acetabulum fractures constitute 2 to 8% of all fractures and result mostly from high-energy trauma. 1 2 Since 1964, with Judet and Letournel, osteosynthesis of deflected acetabulum fractures is believed to ensure better results than conservative treatment. 2 Fractures of two columns of the acetabulum, according to the Letournel classification, are one of the three most common types in frequency, indication, and surgical complexity, with the initial deviation and the presence of intra-articular fragments being the greatest predictors of a less favorable result, and the reduction closer to the anatomical the main predictor of good results. 1 2 3 4 5 6 7 These fractures result essentially from lateral compression mechanisms, being the only type of fracture that reaches the two columns whose fracture traces are above the acetabulum, causing a floating acetabulum, without any part connected to the axial skeleton. 1 4 8 Surgical options include: anterior approach with indirect reduction and fixation of the posterior spine; anterior approach followed by posterior approach, either at the same surgical time (which implies repositioning), or in different surgical times; posterior approach followed by an anterior approach, which is a rare approach, since it should usually start with the anterior route; extended iliofemoral approach, increasingly unadvised because of its high extent and aggressiveness; posterior approach with indirect reduction and fixation of the anterior spine. 2 4 6 8 9 In line with this latter option, the authors present a surgical option (double simultaneous and lateral decubitus approach: Kocher-Langenbeck approach and iliac crest approach) based on the first three clinical cases and their clinical and imaging results.

## Case Report

### Case 1

**Fig. 1:**
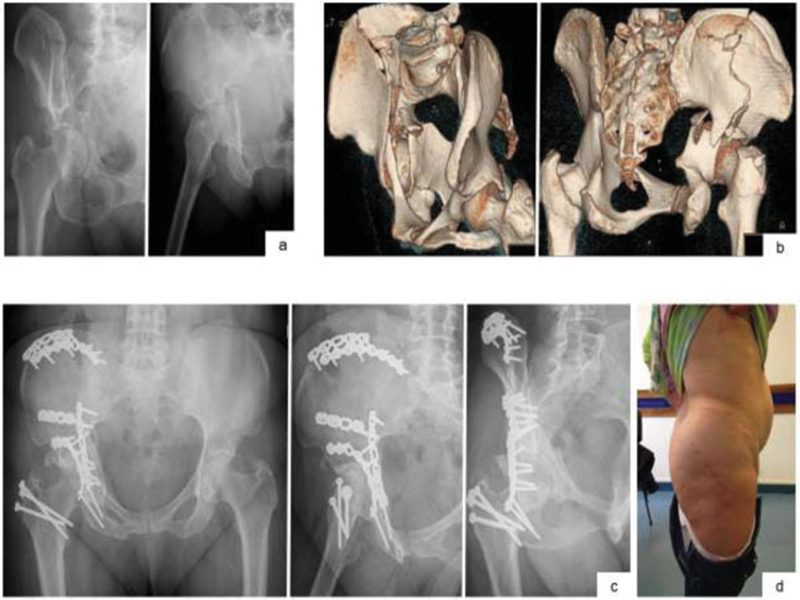
X-ray images at obturator oblique and oblique acetabular views, with typical spur sign on the first image ( **a** ) 3D reconstructions ( **b** ) Radiological control *in inlet, alar* and obturator at the 3rd postoperative year ( **c** ) Photograph of the patient with scars of the routes used ( **d** ).

Female patient, 59 years old, victim of a hit-and-run, arrives at our center with trauma to the right hemi-hip. The imaging study allowed the diagnosis of a fracture of the two columns of the acetabulum with posterior iliac involvement and central dislocation of the femoral head. Skeletal traction was applied to the femoral condits with weight corresponding to 10% of the body weight, and the patient was hospitalized. After surgical planning, progress was made for definitive treatment: reduction and osteosynthesis of the iliac with two plaques and reduction of the posterior spine fracture, and osteosynthesis with plaque via the Kocher-Langenbeck route combined with the iliac crest approach. For better access to the anterior fracture component, fixed with two plates, osteotomy of the large trochanter was required. The postoperative protocol was one commonly used in the institution for this type of lesions and common to the following cases: early initiation of mobilization and muscle strengthening according to scar evolution, discharge of the operated limb during the first 6 weeks and progressive partial load during the following 6 weeks, with total autonomous load allowed at 3 months. With 3 years of evolution, the patient is satisfied, without pain, with autonomous gait and joint mobility similar to the contralateral pelvis, with a Harris Hip Score of 84 points ( Fig. 1 ).

### Case 2

**Fig. 2:**
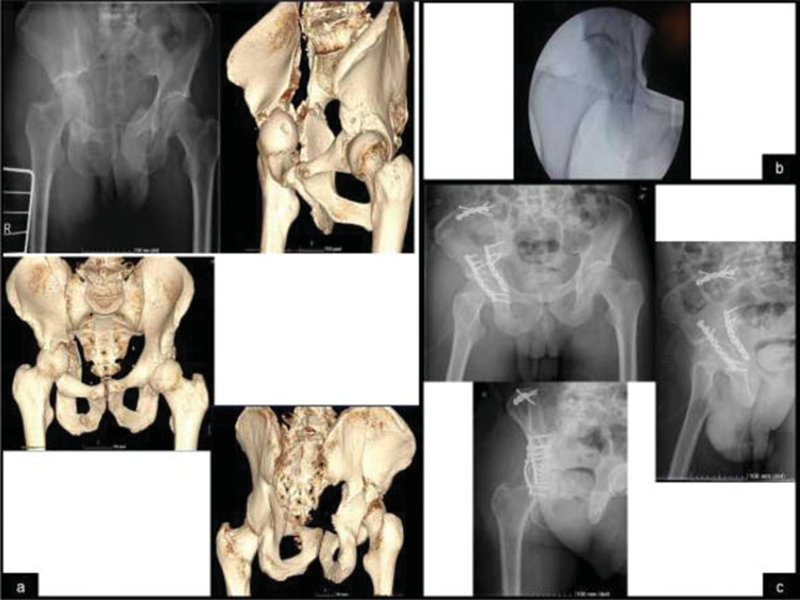
Images of anteroposterior radiography of the pelvis and 3D ACT images for better characterization of the fracture of two columns of the right acetabulum with high extension to the iliac ( **a** ) Intraoperative image after maneuvers to reduce central dislocation ( **b** ) Radiological control in the anteroposterior view of the right pelvis, obturator oblique and oblique acetabular view of the right pelvis in the postoperative period, with acceptable reduction and absence of intra-articular screws.

A 49-year-old male patient was admitted to the emergency department after a 7-meter-high fall with trauma to the right hemi-hip and thoracic trauma, resulting in costal arches fractures with associated pneumothorax and fracture of the two columns of the acetabulum with high extension to the iliac and with central dislocation of the head. After evaluation and multidisciplinary stabilization, we opted for a closed reduction of the central dislocation under anesthesia with fluoroscopic control and application of skeletal traction in the distal femur. After clinical stabilization, definitive surgical treatment was performed: open reduction and osteosynthesis via the Kocher-Langenbeck route combined with the iliac crest approach. The patient complied with the rehabilitation protocol and, at 9 months of evolution, the patient is satisfied and walking without support or limitation in daily life activities, with a Harris Hip Score of 95 points ( Fig. 2 ).

### Case 3

**Fig. 3:**
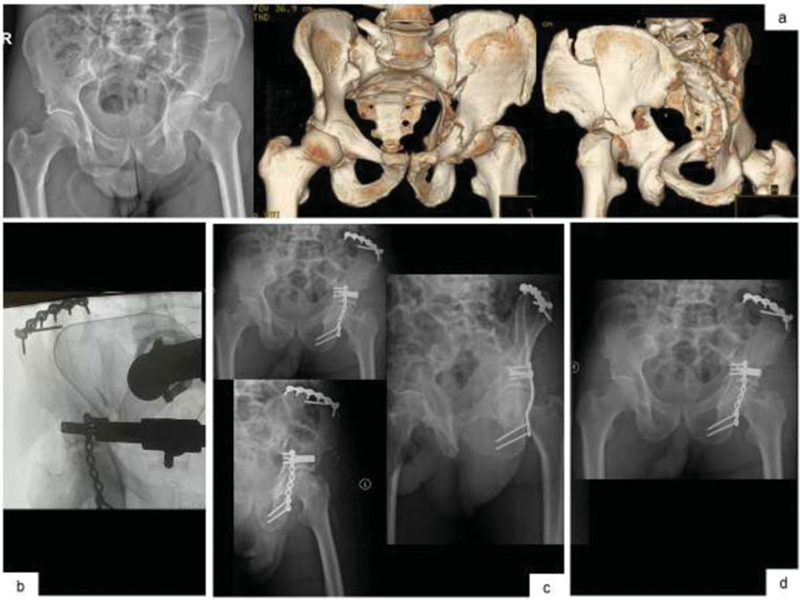
Images of anteroposterior radiography of the pelvis and 3D ACT images for better characterization of the fracture of two columns of the left acetabulum with high extension to the iliac ( **a** ) Intraoperative control image ( **b** ) Radiological control in the anteroposterior view of the pelvis, obturator oblique and oblique acetabular view of the left pelvis in the immediate postoperative period ( **c** ) Anteroposterior radiograph of the pelvis at the 3rd month ( **d** ).

A 69-year-old male patient referred from another institution after a 3-meter-high fall that resulted in a fracture of the two columns of the left acetabulum with high extension to the iliac. After clinical stabilization, complete imaging study and planning, definitive surgical treatment was performed: open reduction and osteosynthesis with posterior spine plate, indirect reduction of the anterior spine and open reduction of iliac fracture, and fixation with two plates and screw in compression via the Kocher-Langenbeck route combined with the iliac crest approach. With 7 months of evolution, the patient presents autonomous gait and is without pain, with a Harris Hip Score of 91 points ( Fig. 3 ).

## Discussion

**Fig. 4:**
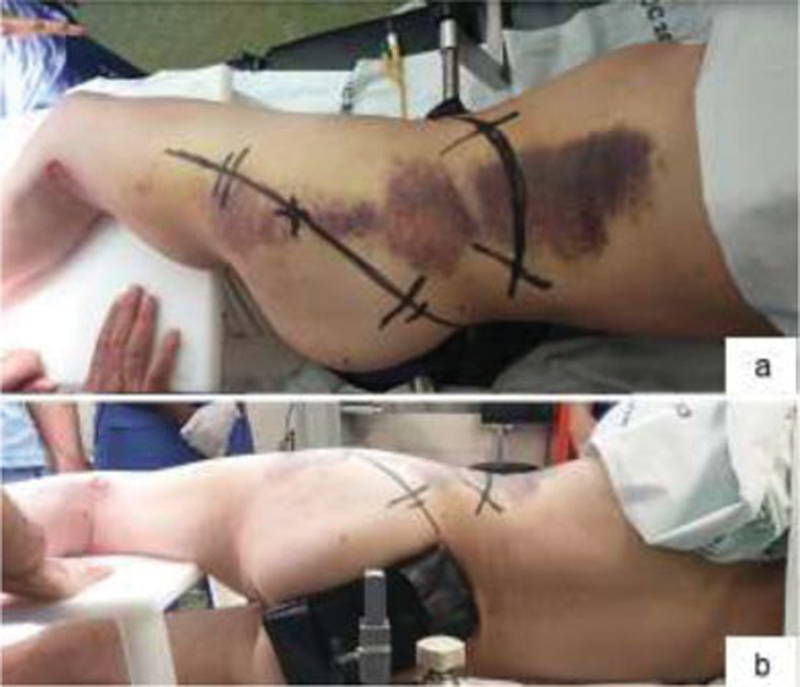
Positioning of the patient in lateral decubitus and prior marking of the incisions (a) Posterior view of the positioning, and the image intensifier enters in this plane ( **b** ).

**Fig. 5:**
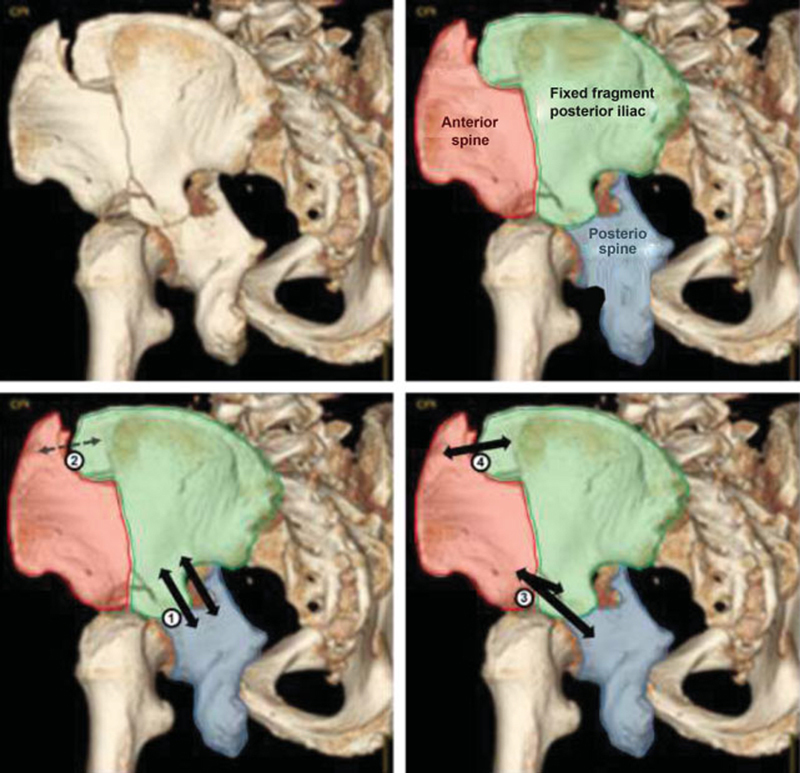
Above: The three main fragments of fractures of two columns of the acetabulum: anterior column, posterior column, and posterior iliac. The latter is connected to the axial skeleton and encompasses the sciatica chamfering; the anterior column (with essentially medial deviation) and the posterior column (with medial and posterior deviation) are free, with their deviation more easily noticeable in the obturator oblique view, where the sciatic chamfering in situ laterally and the medial deviation of the columns and acetabulum (spur sign) are visualized. (6) Below: suggested order of reduction and osteosynthesis of fragments by simultaneous approaches (Kocher-Langenbeck route and iliac crest approach): 1 ^st^ – reduction and fixation of the posterior column to the posterior iliac; 2 ^nd^ – temporary reduction of the upper portion of the anterior spine to the posterior iliac; 3 ^rd^ – reduction of the distal part of the anterior column and its fixation to the posterior iliac and/or posterior column; 4 ^th^ – definitive fixation of the upper portion of the anterior column to the posterior iliac.

Two-column fractures are among the most common acetabular fractures and are characterized as complex and surgically challenging. 1 2 3 4 5 7 The three clinical cases presented in the present case report represent much of what characterizes them: mechanisms of high energy injury, essentially lateral energy vector, and junction of fracture traces above the acetabulum, which is disconnected from the axial skeleton. The surgical decision and planning for patients with this type of lesion requires experience in the different acetabular approaches and implies a deep understanding of the mechanism of injury and diversion of fragments. A detailed evaluation of the patient and of the associated lesions, and a complete imaging study including 3D computed tomography (CT) reconstructions is essential. This planning is the most important step for global success. The authors present three clinical cases in which the characteristics of the lesion dictated the option taken. Fig. 4 shows the positioning and the approach, and Fig. 5 is representative of the suggested reduction and fixation sequence. In the opinion of the authors, the main advantages of this option are: extrapelvic surgery, performed in a single surgical time and positioning, without a potential for complications similar to that of an extended approach. Additionally, in case of intra-articular fragments or associated fractures of the femoral head, it is possible to associate safe pelvis dislocation. In conclusion, despite the need for more forward-looking, randomized, and comparative studies, with relevant samples and follow-up time, the authors believe that the addition of the iliac crest approach to the Kocher-Langenbeck route may be a very attractive option to take into account in the surgical treatment of fractures of two acetabulum columns with greater posterior spine deviation and high extension to the iliac pathway.
