# Supplementary material for: Treatment of Two-column Acetabular Fractures by Double Extrapelvic Approach: Three Clinical Cases
Source: Rev Bras Ortop (Sao Paulo). 2021 Aug 13;59(3):e479–84. doi: 10.1055/s-0041-1729934 (PMC11193574; doi:10.1055/s-0041-1729934)
Supplement: Related texts

# Research article: Tratamento de fraturas acetabulares de duas colunas por dupla via extrapélvica: Três casos clínicos

**Keywords:** acetábulo, fraturas ósseas, pelve

## Abstract

As fraturas de duas colunas do acetábulo segundo a classificação de Letournel são das mais comuns em frequência, indicação e complexidade cirúrgica. Estas resultam essencialmente de mecanismos de compressão lateral e caracterizam-se por originarem um acetábulo desconectado do esqueleto axial. O seu tratamento cirúrgico pode incluir: abordagem anterior ou posterior isolada; combinadas, no mesmo tempo cirúrgico ou não; ou abordagens alargadas. Os autores apresentam outra opção cirúrgica com associação de via de Kocher-Langenbeck e abordagem da crista ilíaca simultânea e no mesmo posicionamento (decúbito lateral) com base nos três primeiros casos clínicos realizados e seus resultados clínicos e imagiológicos. Para além da apresentação dos casos, é feita uma descrição dos três fragmentos característicos deste tipo de fraturas acetabulares, da via de abordagem e da sequência de redução realizada. Pelos resultados obtidos e vantagens associadas, os autores acreditam que a adição da abordagem da crista ilíaca à via de Kocher-Langenbeck pode ser uma opção muito atrativa a ter em conta no tratamento cirúrgico de fraturas de duas colunas do acetábulo devidamente selecionadas.

## Introdução

As fraturas do acetábulo constituem 2 a 8% de todas as fraturas e resultam majoritariamente de traumatismos de alta energia. 1 2 Desde 1964, com Judet e Letournel, se acredita que a osteossíntese de fraturas do acetábulo desviadas garante melhores resultados do que o tratamento conservador. 2 As fraturas de duas colunas do acetábulo, segundo a classificação de Letournel, são um dos três tipos mais comuns em frequência, indicação e complexidade cirúrgica, sendo o desvio inicial e a presença de fragmentos intra-articulares os maiores preditores de um resultado menos favorável, e a redução mais perto da anatómica o principal fator preditor de bom resultado. 1 2 3 4 5 6 7 Tais fraturas resultam essencialmente de mecanismos de compressão lateral, sendo o único tipo de fratura com atingimento das duas colunas cujos traços de fratura se encontram acima do acetábulo, originando um acetábulo flutuante, sem qualquer porção conectada ao esqueleto axial. 1 4 8 Nas opções cirúrgicas, incluem-se: abordagem anterior com redução e fixação indiretas da coluna posterior; abordagem anterior seguida de abordagem posterior, quer no mesmo tempo cirúrgico (o que implica reposicionamento), quer em tempos cirúrgicos distintos; abordagem posterior seguida de abordagem anterior, conduta rara, dado que habitualmente se deve começar pela via anterior; abordagem iliofemoral alargada, cada vez mais desaconselhada pelas suas elevadas extensão e agressividade; e abordagem posterior com redução e fixação indiretas da coluna anterior. 2 4 6 8 9 Na linha desta última opção, os autores apresentam uma opção cirúrgica (dupla abordagem simultânea e em decúbito lateral: via de Kocher-Langenbeck e abordagem da crista ilíaca) com base nos três primeiros casos clínicos e nos seus resultados clínicos e imagiológicos

## Relato de Caso

## Caso 1

Paciente do sexo feminino, 59 anos, vítima de atropelamento, chega ao nosso centro com traumatismo da hemibacia direita. O estudo imagiológico permitiu diagnosticar uma fratura das duas colunas do acetábulo com envolvimento do ilíaco posterior e com luxação central da cabeça femoral. Foi aplicada tração esquelética nos côndilos femorais com peso correspondente a 10% do peso corporal, e a paciente foi internada. Após planeamento cirúrgico, avançou-se para o tratamento definitivo: redução e osteossíntese do ilíaco com duas placas e redução da fratura da coluna posterior e osteossíntese com placa pela via de Kocher-Langenbeck combinada com abordagem da crista ilíaca. Para melhor acesso ao componente anterior da fratura, fixada com duas placas, houve necessidade de osteotomia do grande trocânter. O protocolo pós-operatório utilizado foi o comumente utilizado na instituição para este tipo de lesões e comum aos casos seguintes: início precoce de mobilização e reforço muscular em função da evolução cicatricial, descarga do membro operado durante as primeiras 6 semanas e carga parcial progressiva durante as 6 semanas seguintes, com carga total autônoma permitida aos 3 meses. Com 3 anos de evolução, a paciente encontra-se satisfeita, sem dores, com marcha autónoma e mobilidade articular semelhante à anca contralateral, com um Harris Hip Score de 84 pontos ( Fig. 1 ).

**Fig. 1:** Imagens de radiografias nas incidências obturadora e alar, com típico sinal do esporão na primeira imagem ( **a** ) Reconstruções 3D ( **b** ) Controle radiológico nas incidências *inlet* , alar e obturadora ao 3° ano de pós-operatório( **c** ) Fotografia da paciente com cicatrizes das vias utilizadas ( **d** ).

## Caso 2

Paciente do sexo masculino, 49 anos, deu entrada no serviço de urgência após queda de 7 metros de altura com traumatismo da hemibacia direita e traumatismo torácico, da qual resultaram fraturas de arcos costais com pneumotórax associado e fratura das duas colunas do acetábulo com extensão alta para o ilíaco e com luxação central da cabeça. Após avaliação e estabilização multidisciplinar, optou-se por redução fechada da luxação central sob anestesia com controle fluoroscópico e aplicação de tração esquelética no fêmur distal. Após estabilização clínica, procedeu-se ao tratamento cirúrgico definitivo: redução aberta e osteossíntese pela via de Kocher-Langenbeck combinada com abordagem da crista ilíaca. Foi cumprido o protocolo de reabilitação e, aos 9 meses de evolução, o paciente encontra-se satisfeito e a fazer marcha sem apoio ou limitação para as atividades de vida diárias, com um Harris Hip Score de 95 pontos ( Fig. 2 ).

**Fig. 2:** Imagens de radiografia anteroposterior da bacia e imagens de TAC 3D para melhor caracterização da fratura de duas colunas do acetábulo direito com extensão alta para o ilíaco ( **a** ) Imagem intraoperatória após manobras de redução da luxação central ( **b** ) Controle radiológico nas incidências anteroposterior da bacia, alar e obturadora da anca direita no pós-operatório, com redução aceitável e ausência de parafusos intra-articulares.

## Caso 3

Paciente do sexo masculino, 69 anos, referenciado de outra instituição após queda de 3 metros de altura da qual resultou fratura das duas colunas do acetábulo esquerdo com extensão alta para o ilíaco. Após estabilização clínica, estudo imagiológico completo e planeamento, procedeu-se ao tratamento cirúrgico definitivo: redução aberta e osteossíntese com placa da coluna posterior, redução indireta da coluna anterior e redução aberta da fratura do ilíaco e fixação com duas placas e parafuso em compressão pela via de Kocher-Langenbeck combinada com abordagem da crista ilíaca. Com 7 meses de evolução, o paciente encontra-se a fazer marcha autónoma e sem dores, com um Harris Hip Score de 91 pontos ( Fig. 3 ).

**Fig. 3:** Imagens de radiografia anteroposterior da bacia e imagens de TAC 3D para melhor caracterização da fratura de duas colunas do acetábulo esquerdo com extensão alta para o ilíaco ( **a** ) Imagem de controle intraoperatório ( **b** ) Controle radiológico nas incidências anteroposterior da bacia, alar e obturadora da anca esquerda no pós-operatório imediato ( **c** ) Radiografia anteroposterior da bacia ao 3° mês ( **d** ).

## Discussão

As fraturas de duas colunas são das fraturas acetabulares mais comuns e caracterizam-se por serem complexas e desafiantes do ponto de vista cirúrgico. 1 2 3 4 5 7 Os três casos clínicos apresentados representam muito daquilo que as caracteriza: mecanismos de lesão de alta energia, vetor de energia essencialmente lateral e junção dos traços de fratura acima do acetábulo, que fica desconectado do esqueleto axial. A decisão e planeamento cirúrgico de pacientes com este tipo de lesão exige experiência nas diferentes abordagens acetabulares e implicam um profundo entendimento do mecanismo de lesão e desvio dos fragmentos. É essencial uma avaliação pormenorizada do paciente, das lesões associadas, e estudo imagiológico completo incluindo reconstruções 3D de tomografia axial computarizada (TAC). Este planeamento é o passo mais importante para o sucesso global. Os autores apresentam três casos clínicos nos quais as características da lesão ditaram a opção tomada. A Fig. 4 permite exemplificar o posicionamento e a abordagem, e a Fig. 5 é representativa da sequência sugerida de redução e fixação. Na opinião dos autores, as principais vantagens desta opção são: cirurgia extrapélvica, realizada em um único tempo cirúrgico e posicionamento, sem um potencial de complicações similar ao de uma abordagem alargada. Adicionalmente, em caso de fragmentos intra-articulares ou de fraturas associadas da cabeça femoral, é possível associar luxação segura da anca. Em conclusão, apesar da necessidade de mais estudos prospectivos, randomizados e comparativos, com amostras e tempo de seguimento relevantes, os autores acreditam que a adição da abordagem da crista ilíaca à via de Kocher-Langenbeck pode ser uma opção muito atrativa a ter em conta no tratamento cirúrgico de fraturas de duas colunas do acetábulo com maior desvio da coluna posterior e extensão alta para o ilíaco.

**Fig. 4:** Posicionamento do paciente em decúbito lateral e marcação prévia das incisões ( **a** ) Vista posterior do posicionamento, sendo neste plano que entra o intensificador de imagem ( **b** ).

**Fig. 5:** Em cima: Os 3 fragmentos principais das fraturas de duas colunas do acetábulo: coluna anterior, coluna posterior e ilíaco posterior. Este último fica ligado ao esqueleto axial e engloba a chanfradura ciática; a coluna anterior (com desvio essencialmente medial) e a coluna posterior (com desvio medial e posterior) ficam livres, com o seu desvio mais facilmente perceptível na incidência obturadora, onde se visualiza a chanfradura ciática in situ lateralmente e o desvio medial das colunas e acetábulo (sinal do esporão). (6) Embaixo: ordem sugerida de redução e osteossíntese dos fragmentos por abordagens simultâneas (via de Kocher-Langenbeck e abordagem da crista ilíaca): 1° – redução e fixação da coluna posterior ao ilíaco posterior; 2° – redução provisória da porção superior da coluna anterior ao ilíaco posterior ; 3° – redução da parte distal da coluna anterior e sua fixação ao ilíaco posterior e/ou à coluna posterior; 4° – fixação definitiva da porção superior da coluna anterior ao ilíaco posterior.
